# Classification Performance of Neural Networks Versus Logistic Regression Models: Evidence From Healthcare Practice

**DOI:** 10.7759/cureus.22443

**Published:** 2022-02-21

**Authors:** Richard W Issitt, Mario Cortina-Borja, William Bryant, Stuart Bowyer, Andrew M Taylor, Neil Sebire

**Affiliations:** 1 Clinical Informatics, Great Ormond Street Hospital, National Institute for Health Research (NIHR) Biomedical Research Centre (BRC) University College London (UCL), London, GBR; 2 Statistics, Great Ormond Street Institute of Child Health, University College London (UCL), London, GBR

**Keywords:** performance, neural network, logistic regression, machine learning, clinical informatics, electronic health records

## Abstract

Machine learning encompasses statistical approaches such as logistic regression (LR) through to more computationally complex models such as neural networks (NN). The aim of this study is to review current published evidence for performance from studies directly comparing logistic regression, and neural network classification approaches in medicine.

A literature review was carried out to identify primary research studies which provided information regarding comparative area under the curve (AUC) values for the overall performance of both LR and NN for a defined clinical healthcare-related problem. Following an initial search, articles were reviewed to remove those that did not meet the criteria and performance metrics were extracted from the included articles. Teh initial search revealed 114 articles; 21 studies were included in the study. In 13/21 (62%) of cases, NN had a greater AUC compared to LR, but in most the difference was small and unlikely to be of clinical significance; (unweighted mean difference in AUC 0.03 (95% CI 0-0.06) in favour of NN versus LR.

In the majority of cases examined across a range of clinical settings, LR models provide reasonable performance that is only marginally improved using more complex methods such as NN. In many circumstances, the use of a relatively simple LR model is likely to be adequate for real-world needs but in specific circumstances in which large amounts of data are available, and where even small increases in performance would provide significant management value, the application of advanced analytic tools such as NNs may be indicated.

## Introduction and background

There is increasing interest in the potential role that data science and machine learning can play in healthcare [1-5]. Machine learning encompasses a range of approaches ranging from applied statistical methods of supervised learning such as logistic regression models to more computationally complex models such as various types of neural networks [6].

The aim of applied machine learning methods in healthcare is to improve decision-making and patient management by providing data-based predictions or classifications which are superior to alternative approaches. The majority of decisions in healthcare clinical practice are multifactorial and based on varying degrees of incomplete data. Often, observations on a small number of major risk or predictive features for particular conditions or scenarios are available though they are influenced by biases and medicolegal factors [7]. In this regard, it has been reported in many fields that generally, algorithms or models perform better than human experts, even using simple rule-based systems as well as more advanced algorithms [8]. In medicine, there is increasing interest in the potential value of artificial intelligence and advanced machine learning tools such as neural networks, but such approaches are computationally more expensive and less easily interpretable than simpler statistical methods such as logistic regression models [9].

The purpose of this study is, therefore, to specifically review the current evidence for clinical performance from studies that directly compared logistic regression (LR) and neural network (NN) approaches in medicine in terms of model performance (represented by the area under the curve (AUC) of the receiver operating characteristic (ROC) curve metric) for classification of specific outcomes using identical datasets. The aim is to provide an overview of the current state of published data comparing such methods in order to inform future discussion and strategy relating to health informatics, rather than to provide specific clinical guidance in any medical area, evaluate any specific model, or to compare to the clinical performance of gold standard. We specifically focus on comparing NN and LR as they share a common origin in statistical pattern recognition, and the former may be regarded as a generalisation of the latter [10]. LR is a parametric statistical model; thus, it yields estimates of odds ratios, which allow assessment of the uncertainty of specific aspects of the relationship between the outcome and explanatory variables, and these estimates may be represented as predictions of the outcome variable for specific values of the covariates. NNs are focused on prediction and can be regarded as a fully non-parametric procedure. LR estimates are highly interpretable, which is often not the case with the estimates/parameters of a NN (weights).

## Review

A literature review was carried out in September 2021 on the Pubmed database [11] using the following search terms: (Performance[ti] OR accuracy[ti] OR sensitivity[ti] OR specificity[ti] OR prediction[ti]) AND regression[ti] AND (neural AND network[ti]) OR (tree[ti] OR forest[ti]), all years, English language. Inclusion criteria for selection were primary research studies, which provided information regarding comparative area under the receiver operating curve (AUC) values for the overall performance of both logistic regression (LR) and neural networks (NN) for a defined clinical healthcare-related classification problem using structured data with a categorical output. Articles that examined other machine learning methods, such as decision trees but without LR and NN, were excluded. This was a literature review only, and a Research Ethics Committee approval was not required.

Following an initial search, all potential titles and abstracts were reviewed to remove articles that clearly did not meet the inclusion criteria. The remaining articles were then retrieved, and the abstracts and full texts were examined to determine articles for inclusion in the final list. Performance metrics and key features were then extracted from each article, and the results tabulated. 

**Figure 1:**
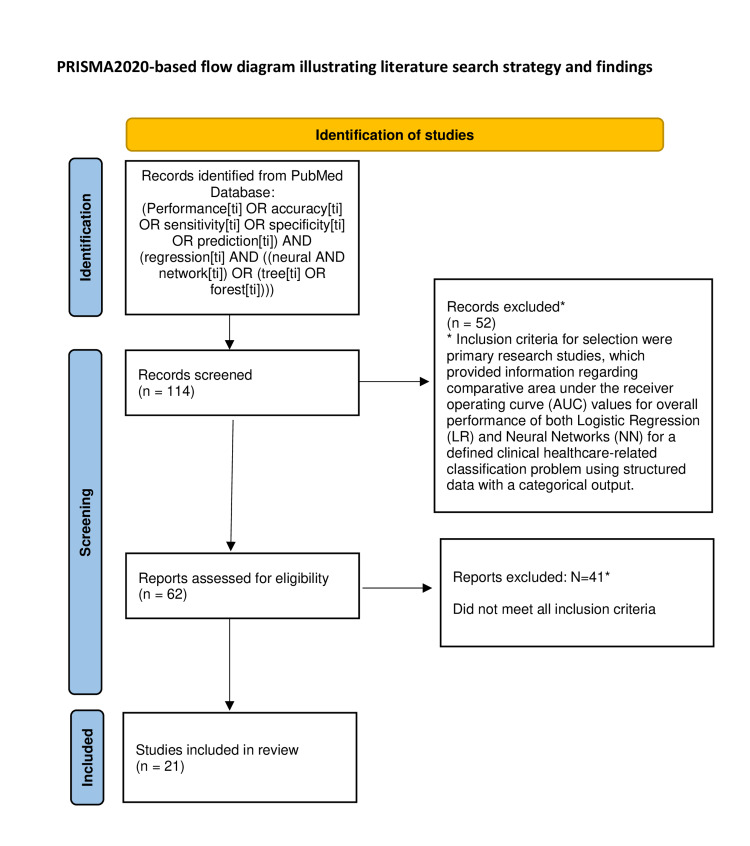
Flow diagram illustrating search strategy and outcome

The initial search revealed 114 articles for potential inclusion. Following title and abstract screening, 62 remained, which after full-text examination resulted in the inclusion of the final 21 studies in which required information was available, including a total of 1,442,703 subjects (Figure 1, Table 1).

**Table 1:** Summary of studies Studies directly comparing the performance of logistic regression (LR) and neural network (NN) machine learning models in clinical medicine, in terms of area under the receiver operating curve (AUC) using identical datasets for specific clinical classification scenarios. (ICU=intensive care unit, SAH=subarachnoid haemorrhage, ED=emergency department)

Author	Clinical area	n	AUC LR	AUC NN
Ing et al. [12]	Giant cell arteritis diagnosis	1,201	0.87	0.86
Kuang et al. [13]	Alzheimer's disease progression	361	0.81	0.9
Owari & Miyatake [14]	Lower back pain progression	96	0.72	0.77
Parsaeian et al. [15]	Low back pain outcome	17,294	0.75	0.75
Abouzari et al. [16]	Subdural haematoma outcome	300	0.59	0.77
Tang et al. [17]	Cardiovascular autonomic dysfunction	2.092	0.76	0.76
Hsieh et al. [18]	Pancreatic cancer diagnosis	1,358,634	0.73	0.61
McLaren et al. [19]	Malignant breast lesion diagnosis	71	0.8	0.82
Lin et al. [20]	ICU mortality	1,496	0.72	0.75
Sakai et al. [21]	Appendicitis outcome	169	0.72	0.74
Erol et al. [22]	Head injury outcome	46	0.9	0.93
Bassi et al. [23]	Survival post cystectomy	369	0.76	0.76
Dumont et al. [24]	Outcome post SAH	91	0.93	0.96
Doig et al. [25]	ICU mortality	422	0.82	0.82
Botha et al. [26]	Structural vascular disease diagnosis	171	0.71	0.71
Borzouei et al. [27]	Thyroid disease diagnosis	310	0.95	0.97
Yao et al. [28]	Diabetic retinopathy diagnosis	530	0.77	0.84
Chen et al. [29]	Hip fracture outcome	10.534	0.88	0.93
Lin et al. [30]	Adipose tissue volume	5,772	0.77	0.9
Tong et al. [31]	Pancreatic cancer outcome	221	0.85	0.92
Sutradhar et al. [32]	Cancer ED visits	42,523	0.67	0.67

**Figure 2:**
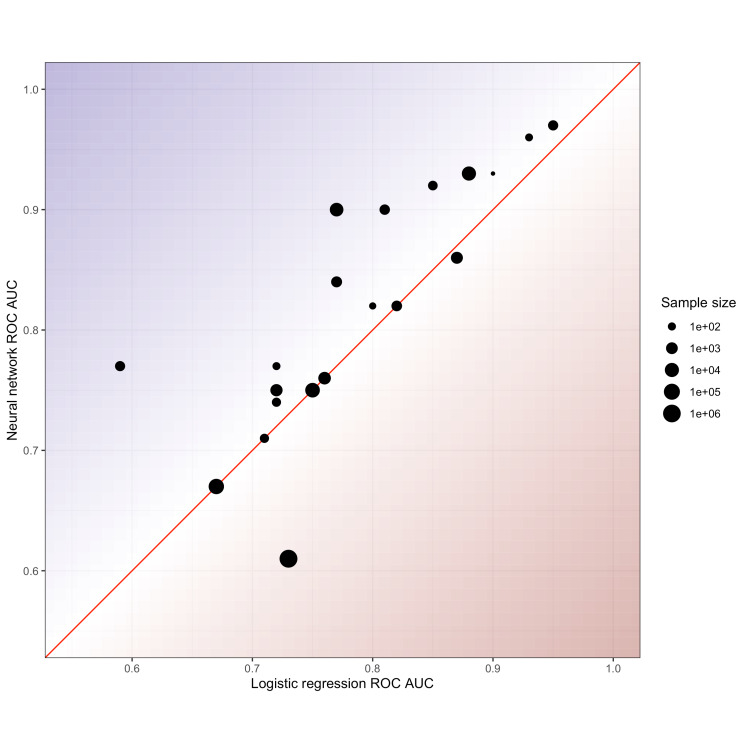
Scatter plot for studies in Table 1 Scatter plot of the area under the receiver operating curve (ROC AUC) results for logistic regression (LR) and neural network (NN) classification models for studies presented in Table 1. The line indicates equal performance, with superior NN performance represented for points above the line and superior LR performance for those below the line. Point size represents study sample size (logarithmic scale). Overall, in the majority of studies, NN performance is similar or superior but the clinical significance remains uncertain.

In 13/21 (62%) of cases, NN had a greater AUC compared to LR, but in almost all cases, the difference was small and unlikely to be of clinical significance; unweighted (large study size heterogeneity) mean difference in AUC 0.03 (95% CI 0-0.06) in favour of NN versus LR (Table 1, Figure 2).

The findings of this study demonstrate that for machine learning performance used in a range of clinical scenarios, complex NNs are slightly superior to simple LR approaches in 60% of cases undergoing direct comparison. However, the overall improvement in performance gained by using NNs is small (mean improvement in AUC of 0.03), and is associated with algorithmic complexity, computational cost and reduced interpretability/ explainability [33]. For specific applications, the trade-off between algorithm performance and costs may justify the use of complex NNs, but in general, the performance of simpler LR based approaches is essentially similar, and at the present time, LR-based machine learning models remain useful initial techniques to address a range of clinical questions.

These findings are consistent with those of the review of machine learning approaches specifically predicting outcome in trauma patients, in which overall, the mean AUC for NN was 0.91 compared to 0.89 with LR [34]. Similarly, in a study of 1,271 patients with a head injury, 1,000 pairs of NN and LR models were run, which demonstrated that in 78% of the trials, AUC for the NN models was superior to the LR model, though in 68% of cases the accuracy of the LR model was superior [35]. Other studies have reported no significant difference in performance, for example, in predicting paediatric meningococcal disease outcome [36]. It should also be noted that in clinical medicine, overall accuracy may not be the correct evaluation metric if the implications of false positive and false negative classification are different and where there is significant class imbalance. However, for the purposes of this study, we simply evaluated one easily compared metric of performance from published studies for two machine learning approaches using the same datasets.

The present study specifically compared LR with NNs, but in a benchmarking study examining 265 datasets from the OpenML repository, which included some biological/ medical datasets, LR performance was compared with that of random forests, another machine learning approach. This comparison reported that overall, random forests performed better than LR in around 70% of datasets, but again the actual difference in performance was small (mean difference 0.04 (95%-CI =0.03-0.05) for AUC), similar to the present findings [37].

In this study, we included studies directly comparing classification performance as assessed by AUC metrics. Whilst this is a reasonable approach to overall performance in this setting, it should be highlighted that AUC may not be a suitable metric for evaluating real-world algorithm performance, particularly in clinical settings in which sensitivity, specificity, false-positive and false-negative rates are differentially weighted in terms of importance. In real-world application evaluation and deployment, a full range of performance metrics should be investigated for any specific model, but for current purposes, we summarise studies that presented a common performance metric, AUC, to allow direct comparison of these algorithmic approaches. Furthermore, the operational or clinical impact and importance of an apparently small incremental improvement in classification performance may vary greatly according to the specific scenario; hence the broad conclusions demonstrated may not necessarily be applicable to all settings. Nevertheless, in most circumstances, there is a trade-off between performance and cost of implementation, with classification algorithms generally intended to provide further evidence for consideration when determining clinical management in the context of numerous other complexities.

In this specific targeted review of medical studies reporting direct AUC performance comparisons, we have not addressed potential issues relating to variable selection/importance of LR models, neuron/layer details of NNs or the appropriateness of using AUC as a marker of discriminatory performance in these specific clinical settings/datasets, since the intention is to provide high-level knowledge regarding the general relative performance of the two approaches, which broadly correspond to examples of ‘white box’ versus ‘black box’ models, rather than specific findings relating to a particular clinical scenario. However, the general methodological similarities and differences between logistic regression and artificial neural network approaches for medical data classification applications have been previously reviewed in detail [10]. Finally, it should be highlighted that whilst LR models may provide acceptable performance compared to NNs for many of the specific applications presented in this review, they are not directly applicable/comparable for more complex machine learning tasks such as image recognition, classification and segmentation, and evaluating details of time-series data such as electrocardiograms, and therefore model selection and approach must be targeted to the specific clinical problem being addressed, and the findings of this review do not necessarily generalise across other medical scenarios.

## Conclusions

The findings of this study demonstrate that in the majority of cases examined across a range of clinical settings, based on classification of categorical outcomes using structured healthcare data, relatively simple LR models demonstrate reasonable performance that is only marginally improved, at the expense of increased complexity, time and computation power, by using more complex methods, in this case, NNs. In many clinical circumstances, the use of such simple LR models is likely to provide adequate performance for real-world needs. However, in specific circumstances, for example, where large amounts of data are readily available, including unstructured data, such as genomic and/or imaging studies, and where even small increases in performance could provide significant management value, development and application of advanced analytic tools such as NNs may be indicated. For most clinical classification and/or prediction problems, a wide range of statistical/machine learning approaches are possible, and relatively quick and accessible techniques such as LR modelling may often be the most appropriate initial technique. Improving performance metrics for machine learning tools in clinical medicine should always be considered, but marginal increases in performance may have limited real-world benefit whilst requiring significantly increased resources and adding complexity. Determining the appropriate method, in addition to the optimal balance of simplicity/complexity versus accessibility/performance for a particular scenario, requires an understanding of both the machine learning methods and practical healthcare implications and highlights the need for the development of multidisciplinary clinical informatics teams in healthcare institutions to address such issues. 
